# Correlation Between Serum Ferritin and Electrocardiographic and Echocardiographic Markers of Cardiac Dysfunction in Children With Transfusion-Dependent β-Thalassemia Major

**DOI:** 10.7759/cureus.116573

**Published:** 2026-09-20

**Authors:** Vipin Kasaudhan, Vijay K Singh, Bhoopendra Sharma, Priyanka Singh, Kuldeep K Singh, Rachna Bhatnagar

**Affiliations:** 1 Pediatrics and Neonatology, Baba Raghav Das Medical College, Gorakhpur, IND; 2 Pediatrics, Baba Raghav Das Medical College, Gorakhpur, IND

**Keywords:** cardiac dysfunction, electrocardiography (ecg), iron overload cardiomyopathy, serum ferritin, trans thoracic echocardiography, β-thalassemia major

## Abstract

Background: Cardiac complications resulting from iron overload remain the leading cause of morbidity and mortality in children with transfusion-dependent β-thalassemia major. Early identification of myocardial dysfunction using non-invasive markers is essential, particularly in resource-limited settings where cardiac magnetic resonance imaging (MRI) T2* is not readily available.

Objectives: To evaluate the correlation between serum ferritin levels and electrocardiographic and echocardiographic markers of cardiac dysfunction in children with β-thalassemia major.

Methods: This analytical cross-sectional study was conducted in the Department of Pediatrics, Baba Raghav Das Medical College, Gorakhpur, India, from January 2025 to December 2025. Eighty children aged two to 16 years with transfusion-dependent β-thalassemia major were enrolled and stratified into three groups according to serum ferritin levels: <2500 ng/mL, 2500-5000 ng/mL, and >5000 ng/mL. All participants underwent clinical evaluation, electrocardiography (ECG), conventional echocardiography, and tissue Doppler imaging. Pearson correlation and multivariate linear regression analyses were performed.

Results: Higher serum ferritin levels were associated with significant prolongation of the corrected QT (QTc) interval and QT dispersion and associated with impairment of left ventricular systolic and diastolic function. Serum ferritin showed significant negative correlations with left ventricular ejection fraction (LVEF) (r = −0.684), E/A ratio (r = −0.582), lateral early diastolic mitral annular velocity (r = −0.658), and tricuspid annular plane systolic excursion (r = −0.512), and positive correlations with the ratio of early transmitral flow velocity to early diastolic mitral annular velocity (E/Ea ratio) (r = 0.726), QTc interval (r = 0.648), and myocardial performance index (r = 0.672) (all p < 0.001). Multivariate analysis identified serum ferritin as the strongest independent predictor of reduced LVEF (β = −0.562, p < 0.001).

Conclusions: Elevated serum ferritin levels are significantly associated with subclinical and overt cardiac dysfunction in children with β-thalassemia major. Combined assessment using serum ferritin, ECG, and echocardiography may facilitate early detection of iron overload cardiomyopathy and help optimize chelation therapy, particularly in settings where advanced cardiac imaging is unavailable.

## Introduction

β-Thalassemia major is one of the most common inherited blood disorders worldwide and remains a major health challenge, particularly in countries of South Asia, the Middle East, and the Mediterranean region. India carries a substantial burden of the disease, with thousands of affected children born every year and a carrier frequency of approximately 3-4% in the general population [1,2]. The disorder results from defective or absent production of the β-globin chain of hemoglobin, leading to severe anemia that requires lifelong regular blood transfusions and iron chelation therapy [3]. Although advances in transfusion practices and chelation therapy have markedly improved survival, repeated blood transfusions inevitably associated with iron accumulation in various organs. Excess iron is primarily deposited in the liver, endocrine glands, and heart. Cardiac iron overload is particularly important because it can lead to myocardial injury, arrhythmias, dilated cardiomyopathy, and heart failure, which remain the leading causes of morbidity and mortality in patients with transfusion-dependent β-thalassemia major [4,5].

One of the major challenges in managing these patients is that cardiac involvement often develops silently. Many children remain asymptomatic during the early stages of myocardial iron deposition, and routine clinical examination may fail to identify early cardiac dysfunction [6]. Therefore, reliable and easily accessible methods for detecting subclinical cardiac involvement are essential so that treatment can be intensified before irreversible myocardial damage occurs.

Electrocardiography (ECG) is a simple and inexpensive tool that can identify abnormalities in cardiac conduction and ventricular repolarization. Parameters such as corrected QT (QTc) interval and QT dispersion have been associated with myocardial iron overload and may indicate an increased risk of arrhythmias in patients with thalassemia [7].

Echocardiography is another widely available, non-invasive method for assessing cardiac function. Conventional measurements, including left ventricular ejection fraction (LVEF), may remain normal until myocardial damage becomes advanced [8]. Tissue Doppler imaging (TDI), however, can detect subtle abnormalities in both systolic and diastolic function at an earlier stage. Parameters such as mitral annular early diastolic velocity (Ea), E/Ea ratio, myocardial performance index (MPI), tricuspid annular plane systolic excursion (TAPSE), and left atrial (LA) functional indices have shown promise in identifying early myocardial impairment before overt heart failure develops [8-10].

Cardiac magnetic resonance imaging using T2* sequences is currently considered the most accurate method for quantifying myocardial iron deposition. However, its availability remains limited in many low- and middle-income countries because of high cost and restricted access. Consequently, serum ferritin continues to be widely used as a practical and inexpensive marker of iron overload in routine clinical practice [5,11-13]. Nevertheless, previous studies have reported inconsistent findings regarding the relationship between serum ferritin levels and cardiac dysfunction [14,15].

Furthermore, information regarding the association between serum ferritin and advanced echocardiographic markers, particularly tissue Doppler parameters and indices of atrial and right ventricular (RV) function, remains limited in children with transfusion-dependent β-thalassemia major [9,10]. Therefore, the present study was undertaken to evaluate the relationship between serum ferritin levels and electrocardiographic and echocardiographic markers of cardiac dysfunction and to identify early indicators of myocardial involvement in this high-risk population.

## Materials and methods

This hospital-based analytical cross-sectional study was conducted in the Department of Pediatrics at Baba Raghav Das Medical College and Nehru Hospital Gorakhpur, Uttar Pradesh, India, from January 2025 to December 2025. Eighty children aged two to 16 years with transfusion-dependent β-thalassemia attending the Pediatric Hematology Clinic or admitted to the Department during the study period were screened for eligibility.

The sample size was calculated for detecting a significant Pearson correlation between serum ferritin levels and echocardiographic markers of cardiac dysfunction using Fisher's Z transformation formula:

n = ((Z_α/2 + Z_β)/C)2 + 3

Where C = 1/2 ln ((1+r)/(1-r))

Assuming an anticipated correlation coefficient of 0.32, a confidence level of 95%, and a study power of 80%, the minimum sample size was estimated to be 74 participants. After accounting for a 10% non-response rate, the final sample size was rounded to 80 children, all of whom were included in the study [16,17].

Eligible participants were selected according to the inclusion and exclusion criteria presented in Table 1.

**Table 1:** Inclusion and Exclusion Criteria HPLC: High-Performance Liquid Chromatography

Inclusion Criteria	Exclusion Criteria
Children aged 2–16 years	Congenital heart disease
Confirmed diagnosis of β-thalassemia major by hemoglobin electrophoresis or HPLC	Acquired heart disease unrelated to iron overload
Receiving regular blood transfusions for at least one year	Acute febrile illness or active infection
Parent/legal guardian provided written informed consent	Chronic kidney disease or chronic liver failure
-	Known thyroid disorders
-	Inadequate echocardiographic window
-	Refusal to participate

To evaluate the effect of increasing iron burden on cardiac function, enrolled participants were categorized according to serum ferritin levels into three groups: Group I (<2500 ng/mL), Group II (2500-5000 ng/mL), and Group III (>5000 ng/mL). These cut-off values were selected based on previous studies evaluating iron overload and cardiovascular complications in patients with β-thalassemia major [14]. Written informed consent was obtained from the parents or legal guardians of all participants before enrolment. After obtaining informed consent, detailed demographic and clinical information was recorded in a predesigned case record form. The following variables were collected: age, sex, age at diagnosis, duration of disease, frequency and duration of blood transfusions, chelation therapy and compliance, history of splenectomy, anthropometric measurements (weight, height, body mass index), pulse rate, and blood pressure.

Laboratory investigations

Three milliliters of venous blood were collected under aseptic precautions for the following investigations: complete blood count, serum ferritin, liver function tests, and renal function tests. Serum ferritin was measured by a chemiluminescent immunoassay method and expressed in nanograms per milliliter (ng/mL).

Electrocardiographic evaluation

All participants underwent a standard 12-lead ECG recorded at a paper speed of 25 mm/s and a calibration of 10 mm/mV. The following ECG parameters were evaluated: heart rate (HR), PR interval, QRS duration, QT interval, QTc, and QT dispersion.

Echocardiographic evaluation

Comprehensive transthoracic echocardiography was performed using a commercially available ultrasound machine (Philips-Affiniti 50; Philips Ultrasound LLC, Bothell, WA, USA) equipped with pediatric transducers by an experienced pediatric cardiologist. Various imaging techniques were performed, including TDI, conventional echocardiography, and pulsed-wave Doppler studies, to evaluate the systolic and diastolic dysfunction of the atria and ventricles using standard parameters. The various parameters were measured according to the recommendations of the American Society of Echocardiography (ASE). LVEF was calculated using the modified Simpson's biplane method [18]. TAPSE was used as a validated and reproducible marker of RV systolic function [19].

Left atrial active emptying fraction was calculated according to standard echocardiographic methodology using the following formula [20]:

LA Active Emptying Fraction (%) = [(Pre-A LA Volume − Minimum LA Volume) / Pre-A LA Volume] × 100

Global myocardial function was evaluated using the MPI [21].

Statistical analysis

Data were entered into Microsoft Excel (Redmond, WA, USA) and analyzed using the SPSS Statistics version 28 (IBM Corp., Armonk, NY, USA). Continuous variables were expressed as mean ± standard deviation (SD) or median (interquartile range), while categorical variables were expressed as frequencies and percentages. The normality of continuous variables was assessed using the Shapiro-Wilk test. Normally distributed continuous variables were expressed as mean ± standard deviation and compared among the three ferritin groups using one-way analysis of variance (ANOVA). Categorical variables were compared using the Chi-square test or Fisher's exact test, as appropriate. Pearson's correlation coefficient was used to assess the relationship between serum ferritin and electrocardiographic and echocardiographic parameters. Before constructing the multivariate linear regression model, the assumptions of linearity, normality of residuals, homoscedasticity, and absence of multicollinearity were assessed and found to be satisfactory. Multiple linear regression analysis was performed to identify independent predictors of LVEF [17]. A two-tailed p-value <0.05 was considered statistically significant.

Ethical considerations

The study protocol was approved by the Institutional Ethics Committee of Baba Raghav Das Medical College, Gorakhpur (Approval No.: 2959(L)/IHEC/2025). Written informed consent was obtained from the parents or legal guardians of all participants prior to enrollment. The study was conducted in accordance with the ethical principles of the Declaration of Helsinki [22].

## Results

Eighty children aged two to 16 years with transfusion-dependent β-thalassemia were included (mean age: 10.31 ± 2.82 years), and 45 (56.3%) were male. Sex distribution did not differ significantly among the three ferritin groups (χ²(2) = 0.086, p = 0.958, Cramer's V = 0.033). Group III children were older and had the longest duration of transfusion therapy (9.71 ± 2.82 years) and the highest annual transfusion frequency (15.62 ± 2.18 transfusions/year) compared with Groups I and II (all p < 0.05). The remaining demographic and clinical characteristics were comparable across the groups (all p > 0.05) (Table 2).

**Table 2:** Baseline Demographic and Clinical Characteristics of the Study Population According to Serum Ferritin Groups *Statistically significant (p < 0.05). Continuous variables are presented as mean ± SD and were compared using one-way ANOVA, F(2,77). Sex distribution was compared using the Chi-square test

Parameter	Group I (<2500 ng/mL) (n=26)	Group II (2500–5000 ng/mL) (n=28)	Group III (>5000 ng/mL) (n=26)	Total (N=80)	p-value
Age (years)	9.42 ± 2.36	10.18 ± 2.84	11.35 ± 2.91	10.31 ± 2.82	0.042*
Male, n (%)	14 (53.8)	16 (57.1)	15 (57.7)	45 (56.3)	0.832
Female, n (%)	12 (46.2)	12 (42.9)	11 (42.3)	35 (43.7)	--
Height (cm)	124.36 ± 12.48	126.82 ± 14.63	129.54 ± 15.27	126.89 ± 14.21	0.156
Weight (kg)	24.18 ± 5.62	25.73 ± 6.84	26.91 ± 7.18	25.61 ± 6.62	0.084
BMI (kg/m²)	15.62 ± 1.84	15.98 ± 2.16	16.03 ± 2.31	15.88 ± 2.09	0.438
Age at diagnosis (years)	1.86 ± 0.94	1.72 ± 0.88	1.64 ± 0.92	1.74 ± 0.91	0.672
Duration of transfusion therapy (years)	7.56 ± 2.18	8.46 ± 2.64	9.71 ± 2.82	8.57 ± 2.68	0.018*
Frequency of transfusion/year	13.42 ± 1.36	14.18 ± 1.84	15.62 ± 2.18	14.39 ± 2.04	0.001*
Age at first transfusion (months)	10.84 ± 4.62	9.96 ± 4.18	9.24 ± 3.96	10.02 ± 4.28	0.146
Duration since last transfusion (months)	2.86 ± 1.12	2.94 ± 1.08	3.12 ± 1.24	2.97 ± 1.14	0.384

The mean serum ferritin level of the study population was 4092.18 ± 2186.42 ng/mL, increasing from 1846.42 ± 486.36 ng/mL in Group I to 6842.36 ± 1246.48 ng/mL in Group III (p < 0.001). Increasing ferritin levels were associated with significantly lower hemoglobin, hematocrit, red blood cell count, and pre-transfusion hemoglobin levels (all p < 0.05). Chelation compliance differed significantly among the groups (χ²(2) = 15.72, p < 0.001, Cramer's V = 0.443), with good compliance declining from 84.6% in Group I to 30.8% in Group III. These findings indicate that greater iron overload is associated with poorer hematological status and reduced adherence to iron chelation therapy (Table 3).

**Table 3:** Hematological and Transfusion-Related Characteristics According to Serum Ferritin Groups Data are expressed as mean ± standard deviation or number (percentage). Continuous variables were compared using one-way analysis of variance (ANOVA), and categorical variables were compared using the chi-square test. Chelation compliance differed significantly among the three groups (χ²(2) = 15.72, p < 0.001, Cramer's V = 0.443). *Statistically significant (p < 0.05).

Parameter	Group I (<2500 ng/mL) (n=26)	Group II (2500–5000 ng/mL) (n=28)	Group III (>5000 ng/mL) (n=26)	Total (N=80)	p-value
Hemoglobin (g/dL)	8.92 ± 0.86	8.64 ± 0.92	8.36 ± 0.94	8.64 ± 0.92	0.032*
Hematocrit (%)	26.84 ± 2.64	25.92 ± 2.84	24.86 ± 2.96	25.88 ± 2.89	0.041*
RBC count (×10⁶/µL)	3.42 ± 0.48	3.28 ± 0.52	3.06 ± 0.56	3.26 ± 0.53	0.042*
Pre-transfusion Hb (g/dL)	8.64 ± 0.82	8.42 ± 0.76	8.18 ± 0.84	8.42 ± 0.82	0.028*
Good chelation compliance, n (%)	22 (84.6)	16 (57.1)	8 (30.8)	46 (57.5)	0.001*
Poor chelation compliance, n (%)	4 (15.4)	12 (42.9)	18 (69.2)	34 (42.5)	---
Serum ferritin (ng/mL)	1846.42 ± 486.36	3642.84 ± 728.62	6842.36 ± 1246.48	4092.18 ± 2186.42	<0.001*

Electrocardiographic abnormalities became more pronounced with increasing serum ferritin levels, with significant differences observed in heart rate, PR interval, QRS duration, QT interval, QT dispersion, and corrected QT interval (all p < 0.05). Heart rate increased significantly across the three groups (F(2,77) = 5.66, p = 0.008, η² = 0.13), and the QTc showed marked prolongation with increasing iron burden (F(2,77) = 24.68, p < 0.001, η² = 0.39). The mean QT interval increased from 342.36 ± 24.18 ms in Group I to 368.42 ± 32.16 ms in Group III (p = 0.008). Likewise, QT dispersion increased significantly from 36.42 ± 8.64 ms to 52.84 ± 12.46 ms (p < 0.001), while the mean QTc interval increased from 408.36 ± 18.42 ms in Group I to 448.62 ± 26.84 ms in Group III (p < 0.001), indicating progressive disturbances in ventricular repolarization with increasing iron overload (Table 4).

**Table 4:** Electrocardiographic Findings According to Serum Ferritin Groups *Statistically significant (p < 0.05). Data are expressed as mean ± standard deviation. Continuous variables were compared using one-way analysis of variance (ANOVA). Degrees of freedom for all ANOVA analyses were F(2,77). Effect sizes are presented as eta-squared (η²). QTc: corrected QT

Parameter	Group I (<2500 ng/mL) (n=26)	Group II (2500–5000 ng/mL) (n=28)	Group III (>5000 ng/mL) (n=26)	p-value
Heart rate (beats/min)	86.42 ± 10.84	92.36 ± 12.46	98.84 ± 14.62	0.008*
PR interval (ms)	142.36 ± 16.42	148.84 ± 18.36	156.42 ± 20.18	0.022*
QRS duration (ms)	78.42 ± 8.36	82.16 ± 9.42	86.84 ± 10.64	0.018*
QT interval (ms)	342.36 ± 24.18	356.84 ± 28.42	368.42 ± 32.16	0.008*
QT dispersion (ms)	36.42 ± 8.64	44.36 ± 10.84	52.84 ± 12.46	<0.001*
QTc interval (ms)	408.36 ± 18.42	426.84 ± 22.36	448.62 ± 26.84	<0.001*

Conventional echocardiographic parameters differed significantly across the three serum ferritin groups. The mean LVEF declined from 64.82 ± 4.36% in Group I to 54.36 ± 6.42% in Group III (F(2,77) = 22.79, p < 0.001, η² = 0.37), while fractional shortening also decreased significantly across the groups (F(2,77) = 23.12, p < 0.001, η² = 0.38). Conversely, stroke volume, cardiac output (F(2,77) = 10.15, p < 0.001, η² = 0.21), and left ventricular mass index (F(2,77) = 23.41, p < 0.001, η² = 0.38) increased significantly with rising ferritin levels. These findings suggest impairment of left ventricular systolic function and ventricular remodeling associated with increasing iron burden (Table 5).

**Table 5:** Conventional Echocardiographic Parameters According to Serum Ferritin Groups *Statistically significant (p < 0.05). Data are expressed as mean ± standard deviation. Continuous variables were compared using one-way analysis of variance (ANOVA). Degrees of freedom for all analyses were F(2,77). Effect sizes are presented as eta-squared (η²).

Parameter	Group I (<2500 ng/mL) (n = 26)	Group II (2500–5000 ng/mL) (n = 28)	Group III (>5000 ng/mL) (n = 26)	p-value
Ejection fraction (EF, %)	64.82 ± 4.36	60.46 ± 5.84	54.36 ± 6.42	<0.001*
Fractional shortening (%)	34.86 ± 3.42	31.42 ± 4.18	27.36 ± 4.62	<0.001*
Stroke volume (mL)	52.36 ± 6.84	56.42 ± 7.42	58.84 ± 8.36	0.028*
Cardiac output (L/min)	4.52 ± 0.64	5.18 ± 0.82	5.76 ± 0.96	<0.001*
Left ventricular mass index (g/m²)	84.36 ± 12.48	96.84 ± 15.36	112.42 ± 18.64	<0.001*

Doppler and tissue Doppler echocardiographic parameters differed significantly across the three serum ferritin groups. Peak E velocity increased significantly (F(2,77)=9.47, p=0.001, η²=0.20), as did peak A velocity (F(2,77)=4.20, p=0.018, η²=0.10), whereas the E/A ratio declined significantly (F(2,77)=8.52, p=0.002, η²=0.18). Tissue Doppler indices also deteriorated significantly, with reductions in S′ velocity, Ea velocity, and the Ea/Aa ratio and an increase in Aa velocity (all p<0.05). Q-S duration and MPI increased significantly, whereas isovolumic acceleration and myocardial velocity gradient declined across the groups (all p<0.05). These findings indicate progressive impairment of diastolic and global myocardial function associated with increasing iron overload (Table 6).

**Table 6:** Doppler and Tissue Doppler Echocardiographic Parameters According to Serum Ferritin Groups *Statistically significant (p < 0.05). Data are expressed as mean ± standard deviation. Continuous variables were compared using one-way analysis of variance (ANOVA). Degrees of freedom for all analyses were F(2,77). Effect sizes are presented as eta-squared (η²). MPI: myocardial performance index

Parameter	Group I (<2500 ng/mL) (n = 26)	Group II (2500–5000 ng/mL) (n = 28)	Group III (>5000 ng/mL) (n = 26)	p-value
Peak E velocity (m/s)	0.86 ± 0.12	0.92 ± 0.14	1.04 ± 0.18	0.001*
Peak A velocity (m/s)	0.58 ± 0.10	0.62 ± 0.12	0.68 ± 0.14	0.018*
E/A ratio	1.48 ± 0.24	1.38 ± 0.28	1.18 ± 0.32	0.002*
S′ velocity (cm/s)	8.42 ± 1.36	7.64 ± 1.42	6.48 ± 1.58	<0.001*
Ea velocity (cm/s)	10.86 ± 1.84	9.42 ± 1.96	7.84 ± 2.14	<0.001*
Aa velocity (cm/s)	7.36 ± 1.42	8.24 ± 1.58	8.96 ± 1.74	0.004*
Ea/Aa ratio	1.48 ± 0.28	1.16 ± 0.32	0.88 ± 0.36	<0.001*
Q-S duration (ms)	242.36 ± 18.42	258.84 ± 22.36	276.48 ± 26.84	<0.001*
Isovolumic acceleration (IVA, m/s²)	1.86 ± 0.42	1.58 ± 0.46	1.26 ± 0.48	<0.001*
Myocardial velocity gradient (MVG, s⁻¹)	4.86 ± 1.24	4.12 ± 1.36	3.36 ± 1.42	0.002*
Tei index (MPI)	0.42 ± 0.08	0.48 ± 0.10	0.56 ± 0.12	<0.001

RV and LA functional parameters differed significantly across the three serum ferritin groups. RV Ea velocity (F(2,77) = 11.84, p < 0.001, η² = 0.24) and RV Ea/Aa ratio (F(2,77) = 20.92, p < 0.001, η² = 0.35) decreased significantly, whereas RV Aa velocity (F(2,77) = 4.18, p = 0.018, η² = 0.10) and RV MPI (F(2,77) = 16.24, p < 0.001, η² = 0.30), indicated progressive RV dysfunction associated with rising ferritin levels. Likewise, LA active emptying fraction (F(2,77) = 13.76, p < 0.001, η² = 0.26) and passive emptying fraction (F(2,77) = 5.42, p = 0.008, η² = 0.12) declined significantly, whereas LA ejection force increased (F(2,77) = 16.88, p < 0.001, η² = 0.31). Overall, these findings indicate progressive impairment of RV and LA function associated with increasing iron burden (Table 7).

**Table 7:** Right Ventricular (RV) and Left Atrial (LA) Functional Parameters According to Serum Ferritin Groups *Statistically significant (p < 0.05). Data are expressed as mean ± standard deviation. Continuous variables were compared using one-way analysis of variance (ANOVA). Degrees of freedom for all analyses were F(2,77). Effect sizes are presented as eta-squared (η²).

Parameter	Group I (<2500 ng/mL) (n = 26)	Group II (2500–5000 ng/mL) (n = 28)	Group III (>5000 ng/mL) (n = 26)	Total (N = 80)	p-value
RV Ea velocity (cm/s)	11.42 ± 2.18	10.18 ± 2.36	8.36 ± 2.54	10.02 ± 2.58	<0.001*
RV Aa velocity (cm/s)	7.86 ± 1.84	8.64 ± 1.96	9.42 ± 2.18	8.64 ± 2.08	0.018*
RV Ea/Aa ratio	1.46 ± 0.28	1.18 ± 0.32	0.92 ± 0.36	1.19 ± 0.38	<0.001*
RV Myocardial Performance Index (RV MPI)	0.36 ± 0.08	0.42 ± 0.10	0.52 ± 0.14	0.43 ± 0.12	<0.001*
LA active emptying fraction (%)	56.48 ± 6.84	51.36 ± 7.42	46.84 ± 8.36	51.56 ± 8.14	<0.001*
LA passive emptying fraction (%)	32.46 ± 5.84	29.84 ± 6.36	26.42 ± 6.84	29.63 ± 6.46	0.008*
LA ejection force (kdynes)	8.64 ± 2.36	10.42 ± 2.84	12.86 ± 3.42	10.63 ± 3.26	<0.001*

Serum ferritin showed a strong negative correlation with LVEF (r = −0.684, p < 0.001), lateral early diastolic mitral annular velocity (Lateral Ea) (r = −0.658, p < 0.001), septal systolic velocity (Septal S′) (r = −0.614, p < 0.001), E/A ratio (r = −0.582, p < 0.001), LA active emptying fraction (r = −0.546, p < 0.001), and TAPSE (r = −0.512, p < 0.001). Conversely, serum ferritin demonstrated significant positive correlations with E/Ea ratio (r = 0.726, p < 0.001), MPI (r = 0.672, p < 0.001), QTc interval (r = 0.648, p < 0.001), and QT dispersion (r = 0.592, p < 0.001). Among all evaluated parameters, the E/Ea ratio exhibited the strongest positive correlation with serum ferritin, whereas LVEF showed the strongest negative correlation (Figure 1).

**Figure 1:**
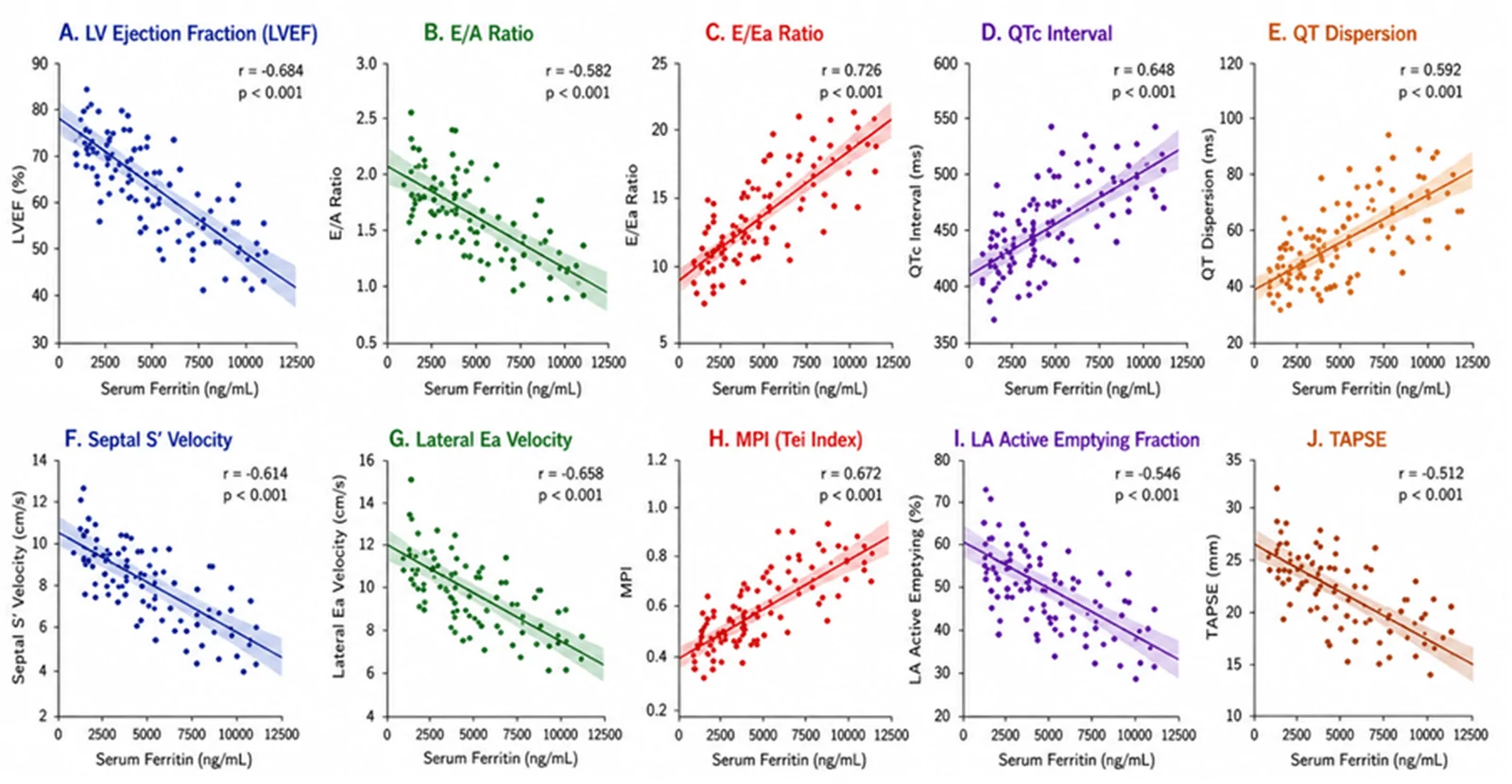
Scatter plots demonstrating correlations between serum ferritin levels and electrocardiographic and echocardiographic markers of cardiac dysfunction. LV: left ventricular, QTc: corrected QT, MPI: myocardial performance index, LA: left atrial

Serum ferritin emerged as the strongest independent predictor of reduced LVEF (β = −0.562, p < 0.001), suggesting that increasing iron overload was significantly associated with worsening left ventricular systolic function. Duration of transfusion therapy was also independently associated with lower LVEF (β = −0.318, p = 0.001). Conversely, good chelation compliance demonstrated a significant positive association with LVEF (β = 0.364, p < 0.001), suggesting a protective effect of adequate iron chelation on cardiac function. Age at diagnosis, transfusion frequency, pre-transfusion hemoglobin levels, age, and gender were not significant independent predictors of LVEF after adjustment for other covariates (p > 0.05 for all). The overall regression model was statistically significant (R² = 0.684, adjusted R² = 0.648, F = 18.96, p < 0.001) (Figure 2).

**Figure 2:**
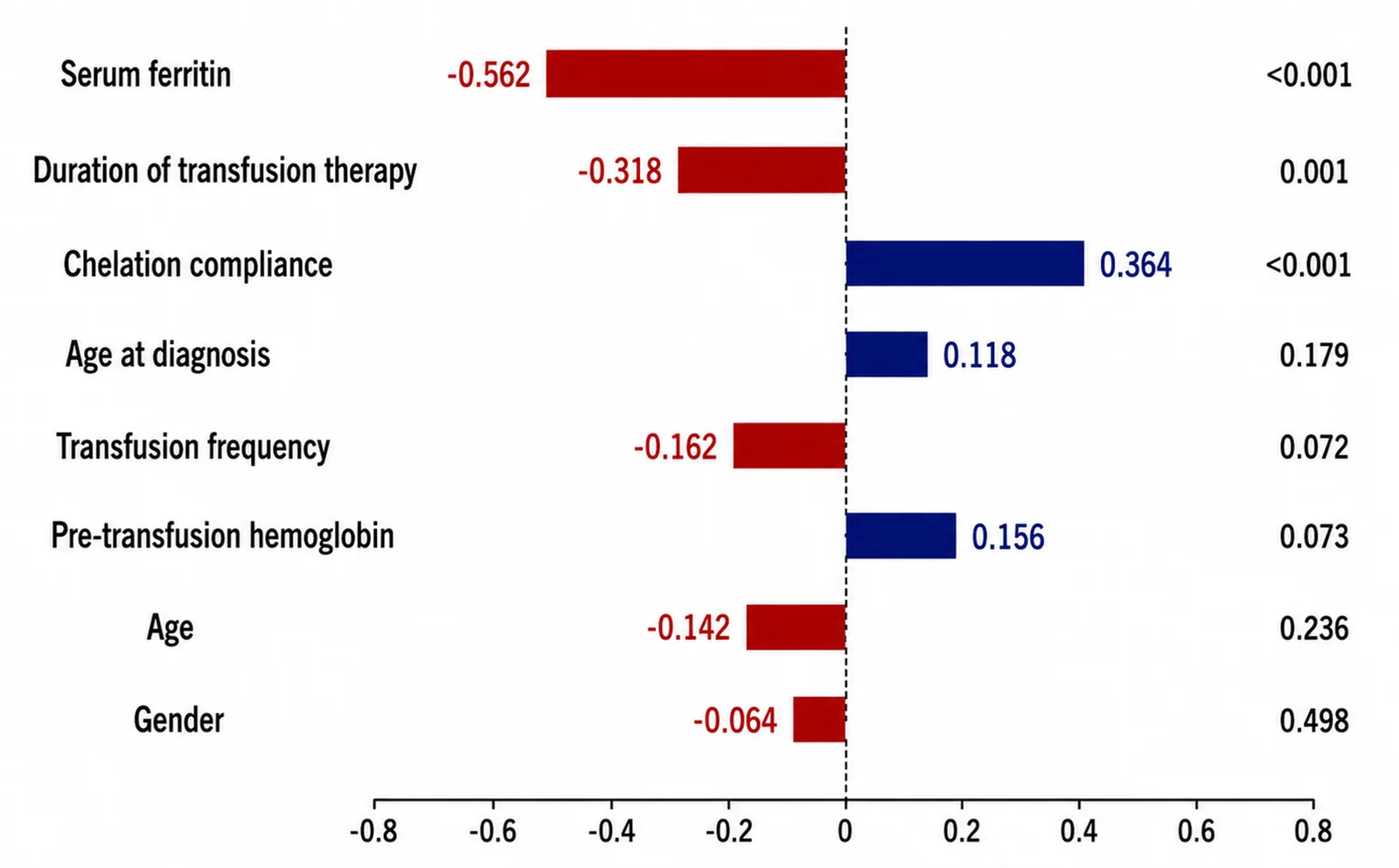
Standardized regression coefficients (β) demonstrating independent predictors of left ventricular ejection fraction in children with β-thalassemia major.

## Discussion

The present study demonstrated a significant association between increasing serum ferritin levels and both electrocardiographic and echocardiographic evidence of cardiac dysfunction in children with transfusion-dependent β-thalassemia major. Serum ferritin was also identified as an independent predictor of reduced left ventricular systolic function.

Despite remarkable improvements in transfusion support and iron chelation therapy, cardiac disease continues to be the major cause of morbidity and mortality in patients with β-thalassemia major [4]. Repeated blood transfusions are associated with iron accumulation within myocardial tissue, resulting in oxidative stress, cellular injury, fibrosis, and eventual ventricular dysfunction [6].

In our study, children with higher ferritin levels had poorer compliance with iron chelation therapy and a longer duration of transfusion therapy. Similar observations were reported by Borgna-Pignatti et al., who demonstrated that effective iron chelation significantly improves survival and reduces the incidence of cardiac complications in patients with thalassemia major [4].

Electrocardiographic abnormalities became more pronounced as serum ferritin levels increased. Prolongation of the QTc interval and increased QT dispersion were particularly evident in children with severe iron overload. These abnormalities reflect disturbances in ventricular repolarization and may predispose patients to potentially life-threatening arrhythmias. Similar findings have been reported by Russo et al., who showed that repolarization abnormalities are common in thalassemia major and are associated with myocardial iron deposition and increased arrhythmic risk [7]. Aessopos and colleagues also observed a higher prevalence of conduction abnormalities and arrhythmias in patients with severe iron overload [23].

Conventional echocardiography in our study demonstrated association of impairment of left ventricular systolic function with increasing ferritin levels. LVEF and fractional shortening declined significantly in children with greater iron burden, whereas cardiac output and left ventricular mass index increased. Eghbali et al. similarly reported impaired ventricular function and increased left ventricular mass among patients with severe iron overload [21].

An important observation in our study was the presence of diastolic dysfunction even before severe systolic impairment became apparent. Children with elevated ferritin levels had significantly lower E/A ratios and reduced tissue Doppler-derived Ea velocities, accompanied by increased E/Ea ratios. TDI has been shown to detect subtle myocardial dysfunction earlier than conventional echocardiographic parameters [8]. Amoozgar H et al. reported comparable findings and demonstrated that reduced Ea velocity and increased E/Ea ratio are early indicators of cardiac involvement in children with β-thalassemia major [10].

We also observed significant reductions in septal S′ velocity, lateral Ea velocity, and isovolumic acceleration among children with higher ferritin concentrations, suggesting increasing evidence of myocardial dysfunction. Moreover, MPI increased significantly with increasing iron burden. Because this index reflects both systolic and diastolic performance, it provides a comprehensive assessment of global ventricular function. Eghbali et al. similarly reported elevated MPI values in thalassemic patients with cardiac iron overload and suggested its usefulness in identifying early myocardial dysfunction [21].

RV function was also adversely affected by iron overload in the present study. Associated reductions in RV Ea velocity, RV Ea/Aa ratio, and TAPSE were observed with increasing ferritin levels, whereas RV MPI increased significantly. Previous investigations have shown that RV dysfunction may occur early in iron overload cardiomyopathy and contribute to exercise intolerance and heart failure [9].

Alterations in LA function were another important finding of our study. Both active and passive LA emptying fractions decreased significantly with increasing ferritin levels, whereas LA ejection force increased. This increase in atrial contractile function may represent a compensatory mechanism in response to impaired ventricular relaxation and elevated filling pressures. Similar alterations in LA mechanics have been described by Blume et al. in patients with diastolic dysfunction and increased ventricular filling pressures [20].

Correlation analysis demonstrated that serum ferritin was significantly associated with several markers of cardiac dysfunction. Strong negative correlations were observed with LVEF, E/A ratio, lateral Ea velocity, LA active emptying fraction, and TAPSE, whereas positive correlations were found with E/Ea ratio, QTc interval, QT dispersion, and MPI. Among these variables, the E/Ea ratio showed the strongest positive correlation with ferritin, while LVEF demonstrated the strongest negative correlation. These findings suggest that association of iron accumulation adversely affects both systolic and diastolic cardiac performance.

Multivariate regression analysis further showed that serum ferritin was the strongest independent predictor of reduced LVEF, even after adjustment for other clinical variables. Duration of transfusion therapy was also independently associated with lower systolic function, whereas good compliance with chelation therapy was associated with better cardiac performance. Similar observations have been reported by Azarkeivan et al., who demonstrated a significant relationship between elevated ferritin levels and impaired cardiac function in patients with β-thalassemia major [14].

The findings of this study have important practical implications, particularly in resource-limited settings where cardiac MRI T2* is not routinely available. Although MRI remains the reference standard for assessing myocardial iron deposition [5,12,24], its cost and limited accessibility restrict its use in many developing countries. Our results suggest that a combination of serum ferritin measurement, ECG, and echocardiography may provide a practical and affordable approach for identifying children at increased risk of iron overload cardiomyopathy and for guiding timely intensification of chelation therapy.

Limitations

This study has several limitations. First, it was a single-center cross-sectional study with a relatively small sample size, which may limit generalizability and preclude inference of causality. Second, serum ferritin levels can be influenced by inflammation, infection, and liver disease and may not accurately reflect myocardial iron deposition.

Finally, advanced echocardiographic techniques, particularly speckle-tracking and strain imaging, were not performed. These modalities may detect subtle myocardial dysfunction earlier than conventional echocardiography and TDI. Therefore, larger multicenter prospective studies incorporating cardiac MRI T2* and advanced echocardiographic techniques are needed to validate our findings and better define the relationship between iron overload and cardiac dysfunction in children with β-thalassemia major.

## Conclusions

In conclusion, higher serum ferritin levels were significantly associated with electrocardiographic and echocardiographic abnormalities, indicating a greater burden of cardiac dysfunction in children with transfusion-dependent β-thalassemia major.

The combined use of serum ferritin, ECG, and echocardiography may help identify early cardiac dysfunction, particularly in resource-limited settings where cardiac MRI T2* is not readily available. Early identification of cardiac abnormalities may facilitate timely optimization of chelation therapy.
